# Demographic and Premorbid Clinical Factors Predict Modified Rankin Score in Large and Medium Vessel Occlusion Ischemic Strokes

**DOI:** 10.3390/jcm14175960

**Published:** 2025-08-23

**Authors:** Tara Srinivas, Dhairya A. Lakhani, Aneri B. Balar, Risheng Xu, Jee Moon, Caline Azzi, Nathan Hyson, Sijin Wen, Cynthia Greene, Janet Mei, Tyler McGaughey, Farzad Maroufi, Jeremy J. Heit, Tobias D. Faizy, Gregory W. Albers, Hamza Salim, Adam A. Dmytriw, Adrien Guenego, Meisam Hoseinyazdi, Vivek S. Yedavalli

**Affiliations:** 1 Department of Radiology and Radiological Sciences, Johns Hopkins University, 600 N. Wolfe St., Phipps B100, Baltimore, MD 21287, USA; 2 Department of Neuroradiology, Rockefeller Neuroscience Institute, West Virginia University, Morgantown, WV 26506, USA; 3 Department of Neurosurgery, Johns Hopkins University School of Medicine, Baltimore, MD 21205, USA; 4 Department of Neuroimaging and Neurointervention, Stanford University, Palo Alto, CA 94305, USA; 5 Department of Radiology, University Hospitals Munster Germany, 48149 Munster, Germany; 6 Department of Neurology, Stanford University, Palo Alto, CA 94305, USA; 7 Department of Neuroradiology, MD Anderson Cancer Center, Houston, TX 77054, USA; 8 Massachusetts General Hospital, Harvard University, Cambridge, MA 02114, USA; 9 Department of Diagnostic and Interventional Neuroradiology, Erasme University Hospital, University of Brussels, 1050 Brussels, Belgium

**Keywords:** ischemic stroke, CTA, outcomes, medium vessel, large vessel

## Abstract

**Background/Objectives:** We report on the association of clinical, demographic, and peri- and intraoperative factors with patient outcomes in large- and, separately, medium-vessel acute ischemic stroke (AIS) occlusions treated with mechanical thrombectomy or medical thrombolysis. Increasingly, neuroimaging, particularly novel markers of collateral status, has become useful in predicting response to endovascular treatment (EVT) among AIS patients. However, the relationship between these neuroimaging markers, documented predictors of stroke outcomes, and post-EVT functional status in anterior circulation large-vessel occlusions (LVOs) as compared to medium-vessel occlusions (MeVOs) remains unclear. We evaluated whether shared predictors of 90-day post-EVT functional outcomes in LVO compared to MeVO AIS patients within our institution exist. **Methods:** We retrospectively evaluated AIS patients treated at our institution between 9 January 2017 and 10 January 2023. The following were the inclusion criteria were applied: (i) CTA confirmed anterior circulation large or medium vessel occlusion; (ii) diagnostic CT perfusion was performed; (iii) mechanical thrombectomy was performed. A low modified Rankin score (mRS) indicating good functional outcomes (i.e., functional independence) was defined as less than or equal to 2, in accordance with prior studies. Univariate and multivariate logistic regression analyses were conducted to determine associations between demographic, clinical, and radiologic factors and mRS ≤ 2. **Results:** A total of 249 LVO (mean age 65.3 ± 16.2, 53.8% female) and 91 MeVO (mean age 68.9 ± 13.3, 46.2% female) patients met the inclusion criteria. Upon multivariate regression adjusted for race, age, hypertension, diabetes mellitus, radiologic features, IV alteplase, admission NIHSS, and reperfusion status, young age (*p* = 0.004), low admission NIHSS (*p* = 0.0001), and good reperfusion status (*p* = 0.007) were associated with good functional outcomes in LVO stroke. By contrast, no factors were significantly associated with good functional outcomes in MeVO stroke. **Conclusions:** Known factors, including young age, low admission stroke severity, and successful reperfusion predict EVT outcomes in LVO stroke but not necessarily in MeVO stroke. Further studies regarding predictors of MeVO outcomes in nonsurgical cases, including collateral status, may guide optimal medical management for this population.

## 1. Introduction

Acute ischemic stroke (AIS) is a leading cause of mortality in the United States [1]. The association between clinical, demographic, and procedural factors and AIS outcomes has been documented; in particular, among M2 medium vessel occlusion (MeVO) strokes, successful recanalization, higher Alberta Stroke Program Early CT Score (ASPECTS), intravenous thrombolysis, age, pre-stroke modified Rankin Scale (mRS), baseline National Institutes of Health Stroke Scale (NIHSS), and diabetes have been identified as outcome predictors [2]. Similar factors have been discussed concerning large-vessel occlusion (LVO) AIS outcomes [3], where LVOs and MeVOs together account for approximately 75% of all AISs [4,5]. However, the relationship between documented outcome predictors, novel imaging markers of collateral status, and functional status after thrombectomy in LVOs and MeVOs remains unclear.

Imaging features that evaluate the collateralization of salvageable tissue surrounding the ischemic core have recently been investigated for their utility in determining the response to endovascular therapy (EVT) for AIS. While there is currently no universally agreed upon marker for pretreatment CT-based collateral status assessment in AIS, several radiologic features have been associated with the reference standard American Society of Interventional and Therapeutic Neuroradiology (ASITN) score upon digital subtraction angiography (DSA) [6,7]. Moreover, a good collateral score as determined by DSA and CTA has been associated with better reperfusion after EVT in large (mostly M1 branch of MCA)-vessel occlusions (LVOs) [1,2,3,4,5,6,7,8,9,10], with variable findings reported on reperfusion outcomes in medium (mostly distal M2 and M3 branches of MCA)-vessel anterior circulation occlusions (MeVOs) [11,12]. Beyond reperfusion, functional outcomes, including the 90-day modified Rankin score (mRS), have been investigated in LVO strokes in relation to collateral status [13,14]; however, less is known about this relationship among MeVO stroke patients, particularly in direct comparison to LVO stroke patients.

Single- and multiphase CT angiography (sCTA and mCTA, respectively) are relatively new measures of collateral status that rely on more rapidly acquired imaging and do not require post-processing in contrast to CTP. Thus, there is increased interest in the use of CTA for collateral assessment to potentially allow earlier interventions in AIS [15,16,17]. Our group has previously shown that CTA markers are associated with DSA ASITN score [7,18], and has developed a scoring system using mCTA derived from CT perfusion source imaging, termed dCTA. Compared to the Tan (which is interpreted on sCTA only) and HIR score (which is a quantitative perfusion metric that represents the volume ratio of Tmax > 10 s volume/Tmax > 6 s volume, thus requiring post-processing), dCTA is interpreted via multiphase imaging and does not require post-processing. Here, we investigate the association of clinical, demographic, and procedural factors, taking into account novel dCTA and other radiologic features, with functional outcomes following thrombectomy in LVO and MeVO strokes within our institution.

## 2. Methods

### 2.1. Study Design

We conducted a retrospective review of patients with acute ischemic stroke (AIS) who were treated at two comprehensive stroke centers at our institution between 9 January 2017 and 10 January 2023. Patients were included based on the following criteria: (1) triage for mechanical thrombectomy occurred within 24 h of symptom onset; (2) pretreatment imaging included noncontrast CT, single-phase CTA (sCTA), and CT perfusion (CTP); (3) CTA confirmed either anterior circulation large-vessel occlusion (including proximal supraclinoid internal carotid artery (ICA), ICA terminus, or M1/proximal M2 segments of the middle cerebral artery, MCA) or anterior circulation medium-vessel occlusion (including distal branches M3 and M4 of the MCA); (4) digital subtraction angiography (DSA) was conducted; (5) mechanical thrombectomy was attempted. Of note, although M1 and M2 occlusions were previously categorized separately due to challenges with the vascular access and reperfusion of M2 lesions, this study classifies M1 and proximal M2 segment occlusions together as large-vessel occlusions (LVOs). This approach aligns with several recent studies that, leveraging technical advancements, have demonstrated comparable mechanical thrombectomy outcomes in M1 and proximal M2 occlusions [19,20,21]. Patients with a premorbid modified Rankin score (mRS) greater than 2, indicating poor functional status, were excluded. The use of IV thrombolysis was determined by the stroke team based on institutional guidelines. This study was approved by the Johns Hopkins Institutional Review Board (IRB00269637) and adhered to the STROBE (Strengthening the Reporting of Observational Studies in Epidemiology) guidelines.

### 2.2. Data Collection

Demographic and clinical data relevant to stroke risk, reperfusion, and outcomes were obtained from electronic health records. These included age, sex, race, ASPECTS, dCTA score, relative cerebral blood flow (rCBF) less than 30% with a volume less than 50 mL, cerebral blood volume (CBV) index, IV tissue plasminogen activator (tPA) administration, pre-stroke mRS, admission NIH Stroke Scale (NIHSS), prior stroke or transient ischemic attack, clinical comorbidities (atrial fibrillation, diabetes, dyslipidemia, hypertension), smoking, blood glucose, occlusion site, modified thrombolysis in cerebral infarction (TICI) score (where mTICI ≥ 2b was considered successful reperfusion), and procedural data (last known well to door time, door to CT time, door to needle time, door to groin time, and groin puncture to recanalization time). The primary outcome was 90d mRS ≤ 2, indicating functional independence and a good outcome.

### 2.3. Imaging Analysis

Imaging was performed using the Siemens Somatom Force CT scanner (Siemens Healthineers, Erlangen, Germany). Dynamic CTA (dCTA) was derived from pretreatment CTP source images, processed using commercial software (RAPID AI v6.1.6, iSchemaView) to generate multiplanar MIP images. CTP was acquired under the following settings: 70 kVP, 200 mAs, a 0.25 s rotation time, an acquisition time of ~60 s, 48 × 1.2 mm collimation, a 0.7 pitch, and a 4D range of 114 mm × 1.5 s. Perfusion maps were generated using RAPID AI software (v6.1.6, iSchemaView) to compute Tmax and CBV values, from which the CBV Index and HIR were derived. The Menon mCTA collateral scoring system was used to assess dCTA, interpreted from the 60 s CTP sequence as a surrogate for multiphase CTA. ASPECTS, mCTA, and sCTA interpretations were independently reviewed by two neuroradiologists (VSY and MH), blinded to clinical outcomes but aware of the occlusion site. Discrepancies were resolved by consensus. Collateral status was graded on ordinal scales; as such, intermodality agreement was assessed using weighted kappa statistics. Continuous agreement analyses (e.g., Bland-Altman) were not employed due to the categorical, non-interval nature of these scoring systems.

### 2.4. Statistical Analysis

Patient characteristics were summarized as either categorical variables (reported as counts and percentages) or continuous variables (means ± SD or medians with interquartile ranges). Bivariate comparisons employed Student’s *t*-test for continuous data, the Mann–Whitney U test for ordinal variables, and Chi-square tests for categorical variables. Spearman correlation was used to evaluate relationships between imaging features and the ASITN score. Logistic regression analyses (univariate and multivariate) were performed to assess associations between predictors and outcomes. Variables with a *p*-value < 0.25 in univariate analysis were included in multivariate models. Subsequently, our data were analyzed in the form of a combined cohort of patients with large-vessel occlusion (LVO) and medium-vessel occlusion (MeVO) strokes. Missing data were addressed using Multiple Imputation by Chained Equations (MICE) with five imputations to minimize bias. A Firth penalized logistic regression model was fitted on each imputed dataset to predict good functional outcome (modified Rankin Scale ≤ 2 at 90 days), adjusting for clinical variables including age, sex, race, vascular risk factors, admission NIH Stroke Scale (NIHSS), intravenous thrombolysis (tPA) administration, imaging scores (ASPECTS, DCTA, DSA), perfusion parameters (rCBF < 30%), and procedural details (mTICI reperfusion grade). The estimates from each imputed dataset were pooled using Rubin’s rules to obtain final effect sizes, confidence intervals, and *p*-values. This approach was chosen to reduce bias from small sample size and separation issues while accounting for missing data. A variance inflation factor (VIF) test for multicollinearity was performed (Supplementary Table S1). A *p*-value ≤ 0.05 was considered statistically significant. All analyses were conducted using R Studio (v4.3.2).

## 3. Results

In total, 249 patients with large-vessel anterior circulation ischemic stroke (mean age 65.3 ± 16.2, 53.8% female) and 91 patients with medium-vessel ischemic stroke (mean age 68.9 ± 13.3, 46.2% female) met the inclusion criteria. The IRR based on kappa agreement for the sCTA was 0.65, the mCTA was 0.68, and the DSA was 0.56 based on independent reviews. Baseline demographic characteristics of LVO and MeVO patients are shown in Table 1. Notably, the dCTA score (*p* < 0.001), DSA score (*p* < 0.001), NIHSS on arrival (*p* < 0.001), median 90d mRS (*p* = 0.02), and groin puncture to recanalization time (*p* < 0.001) were significantly different between the LVO and MeVO groups. In particular, MeVO patients had lower average NIHSSs on arrival and shorter puncture to recanalization times than LVO patients, with a higher median 90-day mRS of three compared to LVO patients, among whom the median mRS at 90 days was two. While DSA was higher in MeVO patients, indicating greater collateral flow, CTA outcome derived from the CT perfusion (dCTA) score was greater in LVO patients. There were no baseline differences in relative cerebral blood flow, <30%, with a volume less than 50 mL (rCBF30% < 50 mL) or in the cerebral blood volume (CBV) index between the LVO and MeVO groups, suggesting no detectable statistical difference in ischemic core and penumbra volume between these groups (Table 1).

Upon univariate analysis, white race was significantly associated with good functional outcomes in both LVO (the odds ratio [OR] for black race with good outcomes, using white race as the baseline, was 0.57, *p* = 0.04) and MeVO (OR 0.37, *p* = 0.02) stroke. Intravenous alteplase administration was also associated with good outcomes in both LVO (OR 2.13, *p* = 0.007) and MeVO (OR 3.60, *p* = 0.007) stroke. As expected, lower NIHSS on arrival was associated with good functional outcomes in both LVO (OR 0.90, *p* < 0.001) and MeVO (OR 0.92, *p* = 0.03). Certain factors, including age (OR 0.96, *p* < 0.001), hypertension (OR 0.37, *p* = 0.002), diabetes (OR 0.40, *p* = 0.002), DSA collateral score (OR 1.48, *p* = 0.001), and successful reperfusion as measured by a mTICI of 2b or greater (OR 4.86, *p* = 0.003) were significantly associated with mRS ≤ 2 in the LVO but not the MeVO group (Table 2). Assessment of multicollinearity was performed using variance inflation factor (VIF) test (Supplementary Table S1). All values were found to be less than the standard evidence-based threshold of 5, suggesting that there are no collinearity issues.

On multivariate regression, young age (aOR 0.96, *p* = 0.003), low NIHSS outcomes on arrival (aOR 0.90, *p* = 0.003), and a mTICI 2b or greater (aOR 7.94, *p* = 0.01) were associated with good functional outcomes in LVO stroke (Table 3 and Supplementary Figure S1). None of these or any other factors found to be significant on univariate analysis were detected as significant upon multivariate analysis of MeVO stroke patients (Table 4), which may have been due to the small sample size leading to low power. Subsequently, sensitivity analysis was conducted (Supplementary Table S2) and a multiply-imputed Firth logistic regression model was employed, which revealed that older age, diabetes, and higher admission NIHSSs were significantly associated with lower odds of achieving a good 90-day outcome (Table 5). Treatment with tPA and achieving successful reperfusion (mTICI of 2b or greater) were strongly predictive of good outcomes. Other factors including sex, smoking, hypertension, and procedural timing variables were not significantly associated after adjustment (Table 5).

## 4. Discussion

In our cohort of 249 patients with large-vessel anterior circulation ischemic stroke and 91 patients with medium-vessel ischemic stroke, young LVO AIS patients with low initial NIHSSs and better thrombolysis in cerebral infarction scores were more likely to achieve good functional outcomes after EVT than their counterparts when adjusting for demographic, clinical, and radiologic factors. Similar findings have been documented previously [22,23,24]. Notably, mTICI ≥ 2b was found to have the highest multivariate odds ratio for good functional outcomes in the LVO cohort, which is consistent with previous large-registry studies [25]. This observation may be due to the fact that, while factors such as race, age and clinical comorbidities influence stroke prognosis, they are non-modifiable at the time of intervention and thus may be less strongly associated with post-procedural outcomes. As such, the clinical importance of angiographically successful reperfusion should be underscored in the consideration of LVO stroke management. There is ongoing important discussion regarding the reasons for and implications of demographic disparities in AIS outcomes. While the adjusted effect of race on functional outcomes was not significant in this cohort, the incidence and prevalence of AIS remain disproportionately high among black and elderly persons [26]. Additionally, little is documented regarding the relationship between demographic characteristics and access to care and social support during the post-stroke recovery period [23]. Ongoing effort is required in the realms of training, advocacy, and research to continue improving parity in AIS care [27].

Predictors of LVO outcomes (young age, low NIHSS admission score) were not observed to be significant among MeVO patients; the evaluation of both the LVO and MeVO cohorts treated within our institution allowed for the comparison of shared predictors with the elimination of variabilities in resource availability. However, upon subsequent sensitivity and bootstrapping analysis of the combined LVO and MeVO cohort, a multiply-imputed Firth logistic regression model revealed that older age, diabetes, and higher admission NIHSSs were significantly associated with worse outcomes, while treatment with tPA achieving successful reperfusion was associated with good outcomes. These analyses suggest that admission NIHSS, age, and successful reperfusion are likely robust and generalizable risk predictors in thrombectomy outcomes, particularly for LVOs. There has recently been discussion regarding the value of EVT for MeVO, with several studies, including the ESCAPE-MeVO trial, DUSK study, and DISTAL trial, finding that EVT does not lead to improved outcomes compared to medical management and may even be associated with increased rates of hemorrhagic conversion and intra- or postoperative complications [11,28]. A conservative treatment approach may be reasonable in select MeVO cases after careful consideration of pretreatment parameters, although further high-powered prospective studies are needed to confirm these observations. In the setting of medical management of MeVO, evaluation of collateral status for clinical guidance and outcome prediction may be increasingly useful.

Notably, in the present study, DSA showed increased collateralization in MeVO compared to LVO patients at baseline, while dCTA showed the opposite trend. This observation may be due to moderately low interrater reliability, which could explain the lack of association between dCTA and functional outcomes in LVO in the present cohort, whereas a significant association has been described in the literature [10,11]. Additionally, MeVO stroke patients have previously been found to have better collateral circulation than LVO stroke patients but may suffer worse outcomes [11,29], suggesting not only that DSA may be a useful tool for collateral assessment in MeVO cases in our cohort but also that further investigation into optimal MeVO management is still needed.

### Limitations

Limitations of this study include its retrospective nature. Cases were sampled from two tertiary care centers at a single institution utilizing one commercial software program; as such, the results may have limited generalizability, particularly to low-resource settings with variable availability of experienced neuroradiologists, interventionalists, and other providers. While the present study did not identify significant predictors of good functional outcomes in MeVO patients, the small number of patients in this group (n = 35 with mRS ≤ 2) limits the statistical power. Additionally, since M3 and M4 occlusions are distal to the primary sources of leptomeningeal collateral supply, the observed differences in collateral status between the LVO and MeVO groups may reflect a selection bias. Although in the present study the interrater reliability for the determination of collateral status using sCTA was 0.65, that using mCTA was 0.68, and that using DSA was 0.56, suggesting the need for greater standardization and/or consistency, particularly in future multicenter studies, there is evidence that variable interrater reliability is common in such CTA-based evaluations [30], which could represent an inherent limitation of the technique.

## 5. Conclusions

This study is novel in reporting the relationship between demographic, clinical, procedural, and neuroradiologic features and functional outcomes in LVO compared to MeVO AIS following EVT within one institution. While young patients with low admission stroke severity and good reperfusion had better 90-day post-treatment outcomes in the LVO cohort, none of these or the other tested factors were significantly associated with good outcomes in the MeVO cohort. Our results suggest that optimal management for LVO may differ from that for MeVO, with the latter potentially benefiting from medical therapy. The role of pretreatment clinical, demographic, and radiologic (e.g., collateral status) factors in MeVO medical management may be elucidated by further investigation.

## Figures and Tables

**Table 1:** Baseline demographic characteristics of LVO (n = 249) and MeVO (n = 91) ischemic stroke patients treated by mechanical thrombectomy.

	LVO	MeVO	
	n (%)	n (%)	*p* Value
**Race (B)**	98 (39.3%)	42 (46.2%)	0.25
**Age, years**	65.3 ± 16.2	68.9 ± 13.3	0.06
**Sex (F)**	134 (53.8%)	51 (56.0%)	0.72
**Hypertension (HTN)**	192 (77.1%)	77 (84.6%)	0.13
**Dyslipidemia**	130 (52.2%)	53 (58.2%)	0.33
**Diabetes mellitus (DM)**	65 (26.1%)	24 (26.3%)	0.97
**Atrial fibrillation**	91 (36.5%)	42 (46.2%)	0.11
**Ever smoking**	116 (46.2%)	37 (40.7%)	0.37
**Periprocedural blood glucose**	135.8 ± 53.2	137.6 ± 64.5	0.79
**Radiologic feature** **ASPECTS** **dCTA** **DSA** **rCBF30% < 50mL** **CBV Index**	N/A 8.6 ± 1.8 **3.2 ± 1.1** **1.9 ± 1.1** 141 (56.6%) 0.8 ± 0.2	N/A 9.0 ± 1.4 **1.9 ± 0.7** **2.7 ± 0.8** 61 (67.8%) 0.8 ± 0.2	N/A 0.06 **<0.001** **<0.001** 0.06 0.59
**IV tPA administered**	90 (36.1%)	30 (33.0%)	0.60
**NIHSS on arrival**	**14.6 ± 7.3**	**11.6 ± 6.3**	**<0.001**
**90 day mRS**	**2 (3)**	**3 (4)**	**0.02**
**Prior stroke or TIA**	50 (20.0%)	12 (13.2%)	0.15
**Occlusion site** **M1** **PM2** **SCICA** **Distal M2** **M3** **M4**	N/A 179 (71.9%) 44 (17.7%) 25 (10.0%) N/A N/A N/A	N/A N/A N/A N/A 86 (94.5%) 7 (7.7%) 2 (1.1%)	N/A
**mTICI** ≥ **2b**	180 (72.8%)	70 (76.9%)	0.45
**Procedural data** **LKW to door, mins** **Door to CT, mins** **Door to needle, mins** **Door to groin puncture, mins** **Groin puncture to recanalization, mins**	N/A 289.8 ± 770.9 46.9 ± 110.3 106.8 ± 234.5 191.8 ± 117.3 **43.9 ± 31.5**	N/A 309.7 ± 419.2 29.4 ± 24.1 66.1 ± 46.5 190.6 ± 172.1 **29.1 ± 19.7**	N/A 0.81 0.13 0.10 0.95 **<0.001**

All data represent the number (% of total), mean ± standard deviation, or median (IQR) unless otherwise indicated. ASPECTS = Alberta Stroke Program Early CT Score; dCTA = CT angiography derived from CT perfusion source imaging; DSA = digital subtraction angiography; rCBF30% < 50 mL = relative cerebral angiography <30% with volume less than 50 mL; CBV = cerebral blood volume; NIHSS = National Institutes of Health Stroke Score; mRS = modified Rankin Score; M1 = first anatomical branch of middle cerebral artery; PM2 = proximal M2 branch of middle cerebral artery; SCICA = supraclinoid internal carotid artery; M3 = M3 branch of middle cerebral artery; M4 = M4 branch of middle cerebral artery; mTICI = modified treatment in cerebral infarction; LKW = last known well.

**Table 2:** Univariate predictors of 90d mRS ≤ 2 in anterior-circulation LVO and MeVO ischemic stroke patients treated by mechanical thrombectomy.

	LVO			MeVO		
	n (%)	OR (95% CI)	*p* Value	n (%)	OR (95% CI)	*p* Value
** Race (B) **	**47 (34.8%)**	**0.57 (0.34–0.97)**	**0.04**	**13 (34.2%)**	**0.37 (0.15–0.87)**	**0.02**
** Age, years **	**60.9 ± 15.7**	**0.96 (0.95–0.98)**	**<0.001**	69.6 **±** 11.1	1.01 (0.98–1.04)	0.64
** Sex (F) **	75 (55.6%)	1.04 (0.63–1.72)	0.86	21 (55.3%)	0.77 (0.34–1.78)	0.55
** Hypertension (HTN) **	95 (70.4%)	**0.37 (0.19–0.70)**	**0.002**	N/A	N/A	N/A
** Dyslipidemia **	69 (51.1%)	0.78 (0.47–1.29)	0.33	N/A	N/A	N/A
** Diabetes mellitus (DM) **	27 (20.0%)	**0.40 (0.22–0.72)**	**0.002**	N/A	N/A	N/A
** Atrial fibrillation **	46 (34.1%)	0.87 (0.45–1.28)	0.31	N/A	N/A	N/A
** Ever smoking **	62 (45.9%)	0.95 (0.57–1.57)	0.84	N/A	N/A	N/A
** Periprocedural blood glucose **	138.8 ± 57.9	1.00 (0.99–1.01)	0.30	126.3 ± 42.7	0.99 (0.99–1.00)	0.17
** Rad features ** **ASPECTS** **dCTA** **DSA** **rCBF30% < 50 mL** **CBV Index**	N/A 1.1 ± 0.9 3.3 ± 1.0 2.2 ± 1.1 81 (59.5%) 0.8 ± 1.1	N/A 1.09 (0.95–1.25) 1.19 (0.89–1.60) **1.48 (1.17–1.88)** 1.91 (0.80–4.55) 5.13 (0.64–41.47)	N/A 0.22 0.23 **0.001** 0.14 0.13	N/A 8.9 ± 1.4 2.0 ± 0.7 2.7 ± 0.8 28 (30.4%) 0.78 ± 0.15	N/A 0.93 (0.71–1.23) 1.17 (0.65–2.11) 1.17 (0.65–2.10) 1.69 (0.15–19.72) 3.66 (0.17–78.21)	N/A 0.62 0.60 0.61 0.67 0.27
** IV tPA administered **	62 (45.9%)	**2.13 (1.23–3.69)**	**0.007**	20 (52.6%)	**3.60 (1.41–9.17)**	**0.007**
** NIHSS on arrival **	**12.6 ± 7.3**	**0.90 (0.86–0.93)**	**<0.001**	10.0 ± 5.7	**0.92 (0.86–0.99)**	**0.03**
** Prior stroke or TIA **	28 (20.7%)	1.12 (0.60–2.10)	0.71	N/A	N/A	N/A
** Occlusion ** **M1** **PM2** **SCICA** **Distal M2** **M3** **M4**	N/A 99 (73.3%) 25 (18.5%) 12 (8.9%) N/A N/A N/A	N/A 0.98 (0.56–1.71) 1.19 (0.62–2.30) 0.79 (0.35–1.81) N/A N/A N/A	N/A 0.94 0.60 0.95 N/A N/A N/A	N/A N/A N/A N/A 35 (92.1%) 2 (5.3%) 1 (2.6%)	N/A N/A N/A N/A 5.20 (0.60–45.05) 0.48 (0.09–2.64) 7.53 × 10^6^ (0-Inf)	N/A N/A N/A N/A 0.14 0.40 0.99
** mTICI ** ≥ **2b**	105 (77.8%)	**4.86 (1.74–13.58)**	**0.003**	35 (92.1%)	2.67 (0.65–10.89)	0.17
** Procedural data ** ** LKW to door, mins ** **Door to CT, mins** **Door to needle, mins** **Door to groin puncture, mins** **Groin puncture to recan, mins**	N/A 348.4 ± 919.4 47.6 ± 129.7 129.8 ± 295.7 197.8 ± 128.9 42.3 ± 31.6	N/A 1.00 (1.00–1.00) 1.00 (1.00–1.00) 1.00 (1.00–1.02) 1.00 (1.00–1.01) 1.00 (0.98–1.01)	N/A 0.26 0.88 0.41 0.49 0.58	N/A 292.6 ± 332.4 29.1 ± 20.0 79.8 6 ± 44.3 161.6 ± 48.1 45.6 ± 24.4	N/A 1.00 (1.00–1.00) 1.00 (0.98–1.02) 1.01 (0.99–1.03) 1.00 (0.99–1.00) 1.00 (0.99–1.02)	N/A 0.36 0.99 0.06 0.19 0.56

All data represent the number (% of total), mean ± standard deviation, or median (IQR) unless otherwise indicated. OR = odds ratio; CI = confidence interval. ASPECTS = Alberta Stroke Program Early CT Score; dCTA = CT angiography derived from CT perfusion source imaging; DSA = digital subtraction angiography; rCBF30% < 50 mL = relative cerebral angiography < 30% with volume less than 50 mL; CBV = cerebral blood volume; NIHSS = National Institutes of Health Stroke Score; mRS = modified Rankin Score; M1 = first anatomical branch of middle cerebral artery; PM2 = proximal M2 branch of middle cerebral artery; SCICA = supraclinoid internal carotid artery; M3 = M3 branch of middle cerebral artery; M4 = M4 branch of middle cerebral artery; mTICI = modified treatment in cerebral infarction; LKW = last known well.

**Table 3:** Multivariate predictors of 90d mRS ≤ 2 in anterior circulation LVO ischemic stroke patients treated by mechanical thrombectomy (n = 249).

	n (%)	OR (95% CI)	*p* Value
** Race (B) **	47 (34.8%)	0.60 (0.25–1.47)	0.26
** Age, years **	**60.9 ± 15.7**	**0.96 (0.93–0.98)**	**0.003**
** Hypertension (HTN) **	95 (70.4%)	0.68 (0.21–2.18)	0.51
** Diabetes mellitus (DM) **	27 (20.0%)	0.53 (0.20–1.42)	0.21
** Rad features ** **ASPECTS** **dCTA** **DSA** **rCBF30% < 50 mL** **CBV Index**	N/A 1.1 ± 0.9 3.3 ± 1.0 2.2 ± 1.1 81 (59.5%) 0.8 ± 1.1	N/A 1.13 (0.88–1.45) 0.88 (0.51–1.52) 1.35 (0.79–2.30) 1.34 (0.29–6.23) 4.62 (0.19–110.99)	N/A 0.33 0.65 0.26 0.71 0.35
** IV tPA administered **	62 (45.9%)	0.99 (0.37–2.59)	0.98
** NIHSS on arrival **	**12.6 ± 7.3**	**0.90 (0.83–0.96)**	**0.003**
** mTICI ** ≥ **2b**	**105 (77.8%)**	**7.94 (1.63–38.68)**	**0.01**

All data represent the number (% of total), mean ± standard deviation, or median (IQR) unless otherwise indicated. OR = odds ratio; CI = confidence interval. ASPECTS = Alberta Stroke Program Early CT Score; dCTA = CT angiography derived from CT perfusion source imaging; DSA = digital subtraction angiography; rCBF30% < 50 mL = relative cerebral angiography < 30% with volume less than 50 mL; CBV = cerebral blood volume; NIHSS = National Institutes of Health Stroke Score; mTICI = modified treatment in cerebral infarction.

**Table 4:** Multivariate predictors of 90d mRS ≤ 2 in MeVO ischemic stroke patients treated by mechanical thrombectomy (n = 91).

	n (%)	OR (95% CI)	*p* Value
** Black race **	13 (34.2%)	0.41 (0.03–5.69)	0.51
** Periprocedural blood glucose **	126.3 ± 42.7	0.99 (0.97–1.01)	0.38
** NIHSS on arrival **	10.0 ± 5.7	0.91 (0.75–1.10)	0.33
** Distal M2 **	35 (92.1%)	1.98 × 10^8^ (0-Inf)	0.99
** mTICI ** ≥ **2b**	35 (92.1%)	0.10 (2.25 × 10^−6^–4378.11)	0.67
** Door to needle, mins **	79.8 6 ± 44.3	1.01 (0.98–1.03)	0.75
** Door to groin puncture, mins **	161.6 ± 48.1	0.99 (0.97–1.01)	0.23

All data represent the number (% of total); mean ± standard deviation; or median (IQR) unless otherwise indicated. OR = odds ratio; CI = confidence interval. NIHSS = National Institutes of Health Stroke Score; mTICI = modified treatment in cerebral infarction.

**Table 5:** Multiply-imputed Firth logistic regression model for predictors of 90d mRS ≤ 2 in combined LVO and MeVO (total n = 340) stroke patients treated by mechanical thrombectomy.

	OR	95% CI	*p*_Value
**Age**	**0.96**	**(0.940–0.982)**	**<0.001**
Sex	1.07	(0.599–1.904)	0.82
Black	0.49	(0.238–1.005)	0.05
Smoking	1.32	(0.736–2.382)	0.35
Hypertension	0.68	(0.306–1.490)	0.33
Dyslipidemia	1.12	(0.597–2.099)	0.73
**Diabetes**	**0.41**	**(0.208–0.818)**	**0.01**
Atrial Fibrillation	0.87	(0.492–1.541)	0.63
**Admission NIHSS**	**0.91**	**(0.870–0.955)**	**<0.001**
**IV tPA**	**2.62**	**(1.358–5.067)**	**0.004**
Preprocedural Blood Glucose	0.99	(0.993–1.004)	0.58
ASPECTS	0.99	(0.793–1.232)	0.92
dCTA	0.76	(0.537–1.068)	0.11
DSA	1.25	(0.853–1.822)	0.25
rCBF30% < 50 mL	2.80	(0.755–10.375)	0.12
CBV Index	2.21	(0.093–52.754)	0.62
Prior Stroke	1.38	(0.652–2.902)	0.40
**mTICI ≥ 2b**	**6.79**	**(1.683–27.404)**	**<0.01**
LKW to door, mins	1.00	(0.999–1.001)	0.43
Door to CT, mins	1.00	(0.993–1.006)	0.89
Door to needle, mins	1.00	(0.995–1.009)	0.53
Door to groin puncture, mins	0.99	(0.996–1.002)	0.64
Groin puncture to recanalization, mins	1.01	(0.983–1.036)	0.50

ASPECTS = Alberta Stroke Program Early CT Score; dCTA = CT angiography derived from CT perfusion source imaging; DSA = digital subtraction angiography; rCBF30% < 50 mL = relative cerebral angiography <30% with volume less than 50 mL; CBV = cerebral blood volume; tPA = tissue plasminogen activator; NIHSS = National Institutes of Health Stroke Score; mRS = modified Rankin Score; mTICI = modified treatment in cerebral infarction; LKW = last known well.

## Data Availability

The data presented in this study are available on request from the corresponding author to maintain patient privacy.

## Supplementary Materials

The following supporting information can be downloaded at https://www.mdpi.com/article/10.3390/jcm14175960/s1. Table S1: Variance inflation factor (VIF) assessment of multicollinearity for multivariable models in large vessel occlusion (LVO) and medium vessel occlusion (MeVO) cohorts. Table S2: Sensitivity analysis of variables predicting good functional outcome (90d mRS ≤ 2) in combined large vessel occlusion (LVO) and medium vessel occlusion (MeVO) cohort using penalized likelihood model. Figure S1: Forest plot for multivariate predictors of 90d mRS ≤ 2 in anterior circulation large vessel occlusion (LVO) and medium vessel occlusion (MeVO) ischemic stroke patients treated by mechanical thrombectomy (n = 249).

## Abbreviations

General Clinical Terms	
AIS	Acute Ischemic Stroke
LVO	Large Vessel Occlusion
MeVO	Medium Vessel Occlusion
EVT	Endovascular Therapy
mRS	Modified Rankin Score (functional outcome measure; ≤2 = good outcome)
NIHSS	National Institutes of Health Stroke Scale (stroke severity score)
TIA	Transient Ischemic Attack
tPA	Tissue Plasminogen Activator (a thrombolytic agent)
IV tPA	Intravenous Tissue Plasminogen Activator
HTN	Hypertension
DM	Diabetes Mellitus
Imaging and Radiologic Metrics	
CT	Computed Tomography
CTA	CT Angiography
sCTA	Single-phase CT Angiography
mCTA	Multiphase CT Angiography
dCTA	CT Angiography derived from CT perfusion source imaging
CTP	CT Perfusion
DSA	Digital Subtraction Angiography
ASPECTS	Alberta Stroke Program Early CT Score (used to assess early ischemic changes)
ASITN	American Society of Interventional and Therapeutic Neuroradiology (used in reference to a standard collateral grading system)
mTICI	Modified Thrombolysis in Cerebral Infarction (reperfusion grading scale; ≥2b = good reperfusion)
CBV	Cerebral Blood Volume
CBV Index	Derived perfusion parameter reflecting penumbra characteristics
rCBF30% < 50 mL	Relative Cerebral Blood Flow <30% with volume less than 50 mL (used to approximate ischemic core)
HIR	Hypoperfusion Intensity Ratio (volume of Tmax > 10 s/volume of Tmax > 6 s)
Tmax	Time to maximum of the residue function, a perfusion parameter
Stroke Anatomy	
MCA	Middle Cerebral Artery
M1	First anatomical segment of MCA
PM2	Proximal M2 segment of MCA
Distal M2/M3/M4	More distal branches of MCA
SCICA	Supraclinoid Internal Carotid Artery
ICA	Internal Carotid Artery
Statistical and Methodological	
IRR	Interrater Reliability
OR	Odds Ratio
CI	Confidence Interval
aOR	Adjusted Odds Ratio
SD	Standard Deviation
IQR	Interquartile Range
Procedural Time Metrics	
LKW	Last Known Well
Door to CT	Time from hospital arrival to CT imaging
Door to needle	Time from arrival to thrombolysis
Door to groin puncture	Time to access for thrombectomy
Groin puncture to recanalization	Time from puncture to reperfusion
