# Supplementary material for: Demographic and Premorbid Clinical Factors Predict Modified Rankin Score in Large and Medium Vessel Occlusion Ischemic Strokes
Source: J Clin Med. 2025 Aug 23;14(17):5960. doi: 10.3390/jcm14175960 (PMC12429499; doi:10.3390/jcm14175960)
Supplement: Supplementary file 1 [file jcm-14-05960-s001.zip › jcm-3714661-supplementary.pdf]

Table S1: Variance inflation factor (VIF) assessment of multicollinearity for multivariable models in large vessel occlusion (LVO) and medium vessel occlusion (MeVO) cohorts.

**Variable**	**VIF**
Black	1.143217791
Age	1.282483697
Hypertension	1.071183079
Diabetes Mellitus	1.052697009
ASPECTS	1.226190474
dCTA	1.218031825
DSA	1.294656235
rCBF30% < 50 mL	1.480679651
IV tPA	1.23672537
Admission NIHSS	1.186787466
mTICI ≥ 2b	1.153166558

ASPECTS = Alberta Stroke Program Early CT Score, dCTA = CT angiography derived from CT perfusion source imaging, DSA = digital subtraction angiography, rCBF30% < 50 mL = relative cerebral angiography < 30% with volume less than 50 mL, tPA = tissue plasminogen activator, NIHSS = National Institutes of Health Stroke Score, mTICI = modified treatment in cerebral infarction.

Table S2: Sensitivity analysis of variables predicting good func-tional outcome (90d mRS ≤ 2) in combined large vessel occlusion (LVO) and medium vessel occlusion (MeVO) cohort using penalized likelihood model.

	**Coefficient**	**Standard error**	**95% CI**	**p**
Age	-0.01443	0.07386	(-0.265-0.259)	0.88
Sex	-0.19651	1.23623	(-5.515-3.218)	0.90
Black	-0.78055	1.562049	(-5.482-2.917)	0.68
Smoking	0.711087	1.868144	(-4.412-8.499)	0.75
Hypertension	-1.22065	2.038591	(-11.264-5.771)	0.62
Dyslipidemia	0.710977	1.725365	(-6.408-9.377)	0.73
Diabetes	-0.3311	1.862944	(-12.900-5.730)	0.87
Atrial Fibrillation	-0.4261	1.559378	(-9.256-4.307)	0.81

Admission NIHSS	-0.0001	0.164913	(-0.462-0.789)	0.99
IV tPA	0.427262	2.179427	(-6.003-8.771)	0.87
Preprocedural Blood Glucose	-0.00242	0.013683	(-0.078-0.045)	0.89
ASPECTS	-0.18764	0.911638	(-5.204-4.097)	0.87
dCTA	0.664785	1.125386	(-2.127-6.140)	0.59
DSA	-0.65371	1.812779	(-6.866-3.588)	0.75
rCBF30% < 50 mL	3.355523	3.260202	(-3.455-16.918)	0.34
CBV Index	-3.69147	5.687984	(-34.304-17.234)	0.59
Prior Stroke	-0.72222	1.836026	(-6.364-6.665)	0.71
mTICI ≥ 2b	-0.11499	3.12259	(-10.466-10.899)	0.98
LKW to door, mins	-0.00776	0.021346	(-0.079-0.107)	0.73
Door to CT, mins	-0.00617	0.053394	(-0.271-0.140)	0.91
Door to needle, mins	-0.02341	0.034644	(-0.134-0.101)	0.53
Door to groin puncture, mins	0.016249	0.01517	(-0.083-0.096)	0.37
Groin puncture to recanalization, mins	0.004634	0.022651	(-0.073-0.099)	0.86

ASPECTS = Alberta Stroke Program Early CT Score, dCTA = CT angiography derived from CT perfusion source imaging, DSA = digital subtraction angiography, rCBF30% < 50 mL = relative cerebral angiography < 30% with volume less than 50 mL, CBV = cerebral blood volume, tPA = tissue plasminogen activator, NIHSS = National Institutes of Health Stroke Score, mRS = modified Rankin Score, mTICI = modified treatment in cerebral infarction, LKW = last known well.

Figure S1: Forest plot for multivariate predictors of 90d mRS ≤ 2 in anterior circulation large vessel occlusion (LVO) and medium vessel occlusion (MeVO) ischemic stroke patients treated by mechanical thrombectomy (n=249).

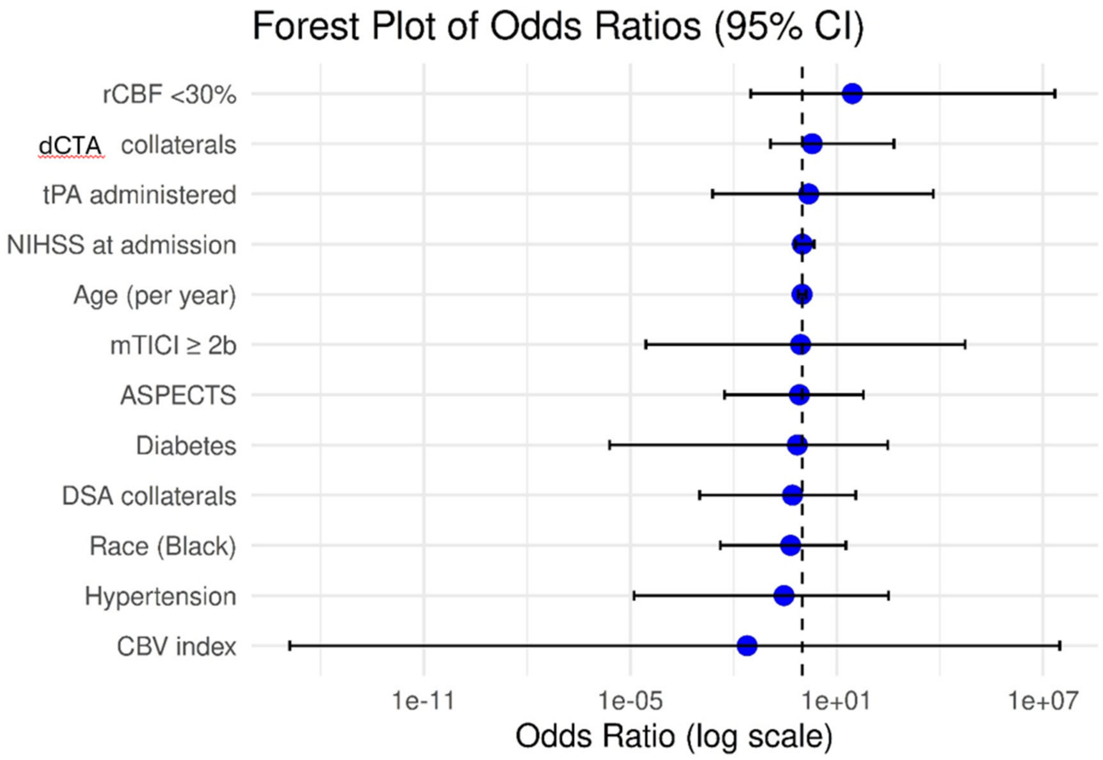

ASPECTS = Alberta Stroke Program Early CT Score, dCTA = CT angiography derived from CT perfusion source imaging, DSA = digital subtraction angiography, rCBF30% < 50 mL = relative cerebral angiography < 30% with volume less than 50 mL, CBV = cerebral blood volume, tPA = tissue plasminogen activator, NIHSS = National Institutes of Health Stroke Score, mTICI = modified treatment in cerebral infarction.
